# Bioaccumulation of ^137^Cs: Vegetation Responses, Soil Interactions and Ecological Implications in the Northern Taiga Ecosystems

**DOI:** 10.3390/life15050774

**Published:** 2025-05-12

**Authors:** Marina Popova, Nikita R. Kriuchkov, Ivan Myasnikov, Alexei Kizeev, Svetlana Ushamova, Dmitriy Manakhov

**Affiliations:** 1 Faculty of Biology, Shenzhen MSU-BIT University, International University Park Road 1, Dayun New Town, Longgang District, Shenzhen 517182, China; 2 Vernadsky Institute of Geochemistry and Analytical Chemistry, Russian Academy of Sciences, ul. Kosygina 19, Moscow 119991, Russia; marbpop@gmail.com (M.P.);; 3 Department of General Ecology and Hydrobiology, Faculty of Biology, Lomonosov Moscow State University, Moscow 119991, Russia; 4 North-West Public Health Research Center, ul. 2nd Sovetskaya 4, Saint-Petersburg 191036, Russia; 5 Lapland State Nature Biosphere Reserve, Murmansk Region, Monchegorsk 184 506, Russia; 6 Department of Radioecology and Ecotoxicology, Faculty of Soil Science, Lomonosov Moscow State University, Moscow 119991, Russia

**Keywords:** radioactive contamination, transfer factor, aggregated transfer factor, northern taiga, Arctic ecosystems, correlation analysis, bioaccumulation, fallout, environmental radioactivity, Kola Nuclear Power Plant

## Abstract

This study presents the first comprehensive examination of ^137^Cs behavior in northern taiga ecosystems of the Kola Peninsula, a previously understudied region regarding radionuclide mobility. The background radioactive contamination of these ecosystems stems from global fallout and differs from more extensively studied contaminated areas. Twelve monitoring sites at varying distances from the Kola nuclear power plant were established to assess ^137^Cs accumulation in dominant plant species across three forest tiers. Gamma-spectrometric measurements revealed high mobility of ^137^Cs with specific activity ranging within 4.7–34.5 Bq/kg in trees, 8.4–164.8 Bq/kg in shrubs, and 15.0–94.5 Bq/kg in mosses. Notably, Ericaceae family plants demonstrated the highest accumulation capacity. ^137^Cs concentrations were significantly higher at the background site (30 km from the power plant) than in the sanitary protection zone, indicating no detectable influence from the nuclear facility. Strong correlations (up to |rs| = 0.93) between bioaccumulation indicators and soil properties were found—particularly with potassium content, exchangeable cation concentration, and organic matter content—suggesting that soil characteristics primarily determine ^137^Cs mobility. These findings highlight the potential risk of ^137^Cs movement through food chains in northern taiga ecosystems, with bioaccumulation coefficients exceeding those of central Russian landscapes and being comparable to those of Scandinavian taiga ecosystems.

## 1. Introduction

Boreal forests, which cover vast areas at the high latitudes of the Northern Hemisphere, play a crucial role in climate regulation, biodiversity conservation, and recreation for the populations of the Nordic countries [1,2]. It is well known that certain areas of Fennoscandia were exposed to radioactive contamination following the Chernobyl Nuclear Power Plant accident in 1986, providing the first insights into how technogenic radionuclides behave in such ecosystems [3,4,5]. Meanwhile, only a few operational radiation-hazardous facilities exist in the region, including the Kola Nuclear Power Plant (Kola NPP), the northernmost nuclear power plant in Europe.

Additionally, nuclear weapons tests conducted in the 1960s led to the accumulation of ^137^Cs in local soils due to global fallout, with contamination densities at polar latitudes being higher than at mid-latitudes [6,7,8]. However, studies on the behavior of ^137^Cs in northern taiga ecosystems under background conditions remain scarce. These forests are located thousands of kilometers north of the regions affected by major radiation incidents in the past and differ from them in climatic conditions, soil properties, and zonal vegetation. Thus, it is questionable whether all the patterns observed, for instance, in studies of the Chernobyl Exclusion Zone or the contaminated territories of Russia and Belarus, are applicable to the northern taiga [9,10,11].

Our research on the behavior of ^137^Cs in the soils of the Kola Peninsula has revealed its remarkably high mobility under northern taiga conditions. Although cesium activity was the highest in forest litter, the underlying mineral horizons contributed significantly to soil contamination density [12]. Its distribution among different chemical forms demonstrated that a relatively large proportion of this radionuclide exists in water-soluble and exchangeable forms in albic podzols [13]. Furthermore, we found that Cladonia lichens actively accumulate cesium [14]. However, while lichens are part of vegetation, they lack a root system and interact with the soil only indirectly.

Our research, along with literature data and public reports from the Kola Nuclear Power Plant on environmental safety, does not indicate a significant impact of the nuclear power plant on the radioactive contamination of the soil and vegetation cover [15,16,17,18]. Nevertheless, due to the nutrient-poor nature of podzols and other soil characteristics, ^137^Cs exhibits high mobility in albic podzols [12].

In this study, we continue to investigate the behavior of ^137^Cs in the soil and vegetation cover of northern taiga ecosystems, using the Kola Nuclear Power Plant impact zone as an example. For the first time, we examine how cesium accumulates in plant parts across a wide range of dominant species from the three vertical layers of boreal forests—trees, shrubs, and mosses—under background contamination conditions.

## 2. Materials and Methods

### 2.1. Study Area and Monitoring Sites

**Figure 1:**
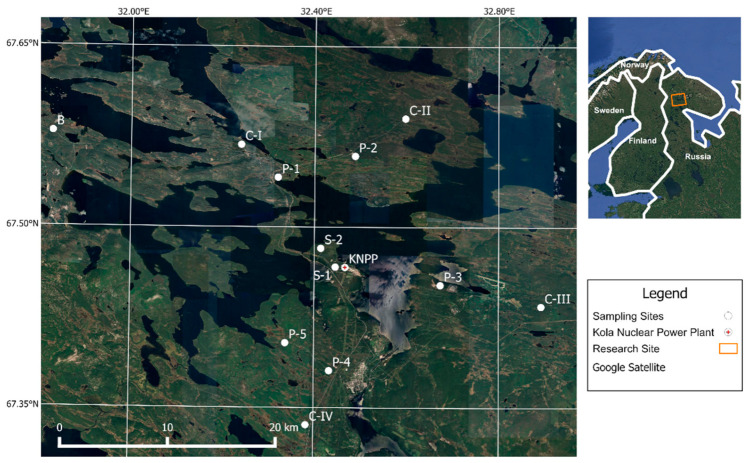
Research site. One site is located within the sanitary protection zone (SPZ) of the nuclear power plant—S-1; Six test sites are in the observation zone (OZ), at a distance of up to 10 km from the station—S-2, P-1, P-2, P-3, P-4, and P-5; Four test sites are on the OZ border at a distance of 15 km—C-I, C-II, C-III, and C-IV; One background site is located at a distance of 30 km from the station—B [12].

This study was conducted in 2014 on a network of monitoring sites in the form of a radial–concentric system consisting of 12 stationary sites (Figure 1): 

Automorphic areas with homogeneous vegetation were chosen for the sites. The studied sites are located beyond the Arctic Circle in the Murmansk region in the area of Lake Imandra. The dominant type of forest communities in the research area are blueberry–lichen–pine forests [15]. The geobotanical descriptions of all sites are given in Supplementary File S1 and Appendix A.

### 2.2. Vegetation and Soil Characteristics

The soils are mainly represented by albic podzols [19], and the soil-forming rocks are sandy moraine deposits. 

The stands are dominated by Scots pine (*Pinus sylvestris* L.) of the V and Va class of boniteness. There are also silver birch (*Betula pendula Roth. X*), fluffy birch (*Betula pubescens Ehrh.*), and Siberian spruce (*Picea obovata Ledeb.*). The grass and shrub layer is dominated by representatives of the genus Vaccinium—European blueberry (*Vaccinium myrtillus* L.), as well as lingonberry (*Vaccinium vitis-idaea* L.). There are rosemary (*Ledum palustre* L.), crow’s feet (*Empetrum hermaphroditum Lange ex Hagerup*), and northern bilberry (*Vaccinium uliginosum* L.). The lower tier is formed by lichen cover, which is dominated by lichens of the genus *Cladonia*—*C. stellaris*, *C. rangiferina* L., and *C. alpestris*. These are bushy, highly branched ground-based (epigeal) lichens typical of the northern taiga forests. In some cases, the lower layer on the sites is a moss–lichen cover formed by lichens and green mosses, among which *Pleurozium schereberi* and *Hyloconium splendens Hedw.* occupy a dominant position [14].

Samples of Scots pine and European blueberry were collected from each site. Samples of other plant species were also collected if they grew on the site in large quantities. Thus, samples of birch and bilberry were collected at all sites, rosemary at 11 sites, Schreber’s moss at 9 sites, and spruce at 7 sites. Subsequently, the samples of pine and spruce were split into branches and needles; birch, blueberry, bilberry, and rosemary were split into branches and leaves. 

### 2.3. Sampling and Analytical Methods

Samples were collected in close proximity to the soil profile. A total of 130 plant samples were included in this study. After harvesting, they were dried at a temperature of 105 degrees and ground to a homogeneous state. The mass of oven-dried plant samples ranged from 100 to 800 g. The specific activity of ^137^Cs in all samples was determined gamma-spectrometrically on the “Multirad” complex with the NaI(Tl) 63 × 63 scintillation gamma detector with Progress software (NTC Amplitude Ltd., Russia) in five-fold repetition with an exposure of 10,800 s. 

### 2.4. Data Analysis and Calculations

For plant parts, the transfer factors (*TF*) of ^137^Cs were calculated as

(1) $$TF=\frac{A_{plant}}{A_{soil}},$$

where *A_plant_* is the specific activity of ^137^Cs in a plant or part of it (Bq/kg) and *A_soil_* is the weighted average specific activity of ^137^Cs in the root column, which amounts to the upper 30 cm layer of soil (Bq/kg).

The aggregated transfer factors (*TFag*) were also calculated:

(2) $$TFag=\frac{A_{plant}}{A_{reserve}},$$

where *A_plant_* is the specific activity of ^137^Cs in a plant or part of it (Bq/kg) and *A_reserve_* is the contamination density (reserve) of ^137^Cs in the 0–30 cm soil layer (Bq/m^2^) [20].

The measurement uncertainty (specific activity of ^137^Cs in all samples) was calculated as the standard deviation of the counting rate. The uncertainty of indirect measurements (contamination density of ^137^Cs, *TF*, and *TFag*) was calculated as the combined standard uncertainty of the entire individual uncertainty of the components [21]. 

Basic descriptive statistics were calculated for all specific activities, *TF*, and *TFag*, and we performed a test for normality by using the Shapiro–Wilk test. To find significant differences between the values of these indicators at the studied sites, a comparison was carried out by using the *t*-test, and if the distribution did not correspond to the normal one, a comparison was carried out by using the Kruskal–Wallis test. To establish possible relationships among the accumulation of ^137^Cs in plant parts, soil properties, and variables indicating the possible effect of Kola NPP on the bioaccumulation of ^137^Cs, a correlation analysis was performed with the calculation of Spearman rank correlation coefficients. 

In the study, long-term data on the speed and frequency of winds in the area of the nuclear power plant were also used (for the period 2012–2016). They were obtained from Kola NPP meteorological stations. Data on soil properties and the vertical distribution of ^137^Cs in soils were taken from our previous study [12].

## 3. Results

### 3.1. Radiocesium Accumulation

**Figure 2:**
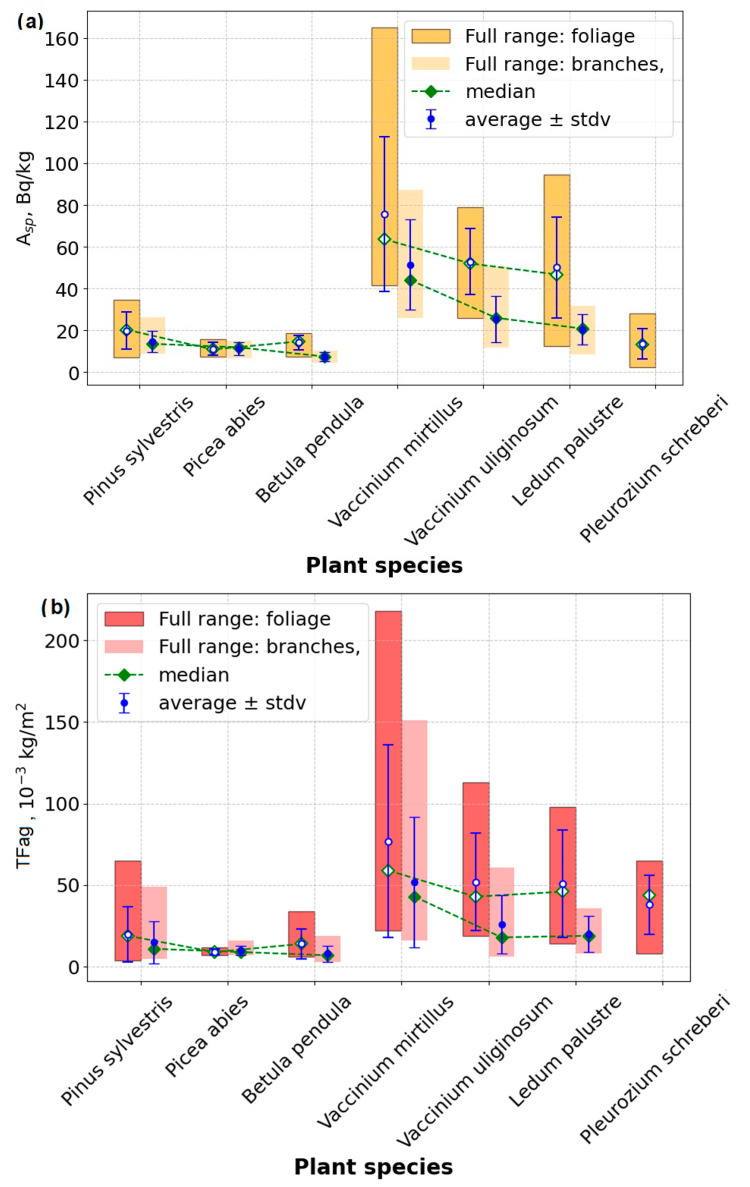

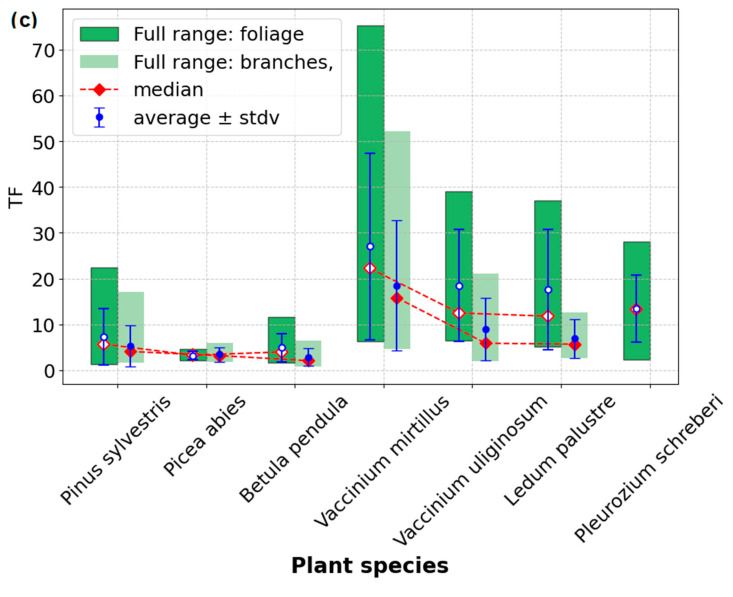
Radiocesium dynamics in vegetation parts across ecosystem layers in foliage and branches (more transparent) of studied species: Tree layer: *Pinus sylvestris* (n = 12)*, Picea abies* (n = 7), and *Betula pendula* (n = 12). Understory layer: *Vaccinium mirtillus* (n = 12)*, Vaccinium uliginosum* (n = 12), and *Ledum palustre* (n = 11). Mosses (*Pleurozium schreberi*, n = 9) are shown as a single column due to undifferentiated structure: (**a**) specific radioactivity of ^137^Cs Bq/kg; (**b**) TFag (the aggregated transfer factors 10–3 kg/m^3^); (**c**) TF (transfer factors, dimensionless quantity). Moss data: represented as whole organisms (no compartmentalization).

The distribution of specific radioactivity (Figure 2a) and the bioaccumulation indicators—*TFag* and *TF* (Figure 2b and Figure 2c, respectively)—illustrates the values for the studied species. Mean values with standard deviations are represented by blue markers with error bars, medians are shown as green and red dashed lines, and value ranges are depicted as rectangles. The outlined rectangles refer to leaves and fir needles, while less contrasted rectangles denote plant branches; therefore, no values for mosses are included in this category.

The specific activities of ^137^Cs in the tree-layer plant parts ranged from 4.7 to 34.5 Bq/kg, in the shrub layer from 8.4 to 164.8 Bq/kg, and in the moss cover from 15.0 to 94.5 Bq/kg. The specific activities for all plant parts are provided in Supplementary File S2. Leaves generally contained more cesium than branches, which was confirmed by comparing the mean specific activity values in branches and leaves of each plant by using the *t*-test and the Wilcoxon signed-rank test for dependent variables (Supplementary File S5).

### 3.2. Bioaccumulation Indicators

The calculation of the bioaccumulation indicators—*TFag* and *TF*—demonstrated that all dominant plant species functioned as accumulators of radiocesium from the soil, with mobility in the soil–plant system being notably high (Supplementary File S2). The *TF* values in the plant parts reached up to 75, while the *TFag* values were as high as 218 kg/m^2^. The highest accumulation levels were observed in Ericaceae plants. The shrub layer accumulated ^137^Cs more intensively than the tree layer.

The series representing the increasing migratory mobility of ^137^Cs, based on the calculation of *TFag*, *TF*, and specific activity, are presented in Figure 2a, Figure 2b, and Figure 2c, respectively.

### 3.3. Accumulation Patterns

The calculation of *t*-tests and H-tests showed that as the distance from the nuclear power plant decreased, cesium accumulation in vegetation did not become more intensive (Table 1). On the contrary, at the background site, the values of specific activities, *TFag*, and *TF* were generally significantly (*p* < 0.05; t_critical = 2.26) higher than those in the sanitary protection zone (Supplementary File S3). This suggests that no influence of the Kola NPP on cesium accumulation in vegetation was detected, and the same was true for the lichen cover [12] and the soils [14]. 

The correlation analysis revealed no significant relationships between the indicators of ^137^Cs bioaccumulation in plant parts and the variables indirectly reflecting the potential influence of the regular operation of the Kola NPP on ^137^Cs deposition in the vegetation cover, namely, the distance from the Kola NPP to the sampling site, the altitude above sea level, and local wind speed and frequency.

Meanwhile, the correlation analysis revealed significant relationships between bioaccumulation indicators (specific activity, *TFag*, and *TF*) and several soil properties, primarily with potassium content, exchangeable cations (Ca^2^⁺, Mg^2^⁺), and organic matter and litter storage (Supplementary File S4; Table 2).

In some cases, significant correlations were also identified for hydrolytic acidity (specifically for *Vaccinium uliginosum* bioaccumulation indicators) and for soil physical clay content (specifically for *Ledum palustre* bioaccumulation indicators).

## 4. Discussion

The conducted study represents the first attempt to investigate ^137^Cs behavior under radioactively uncontaminated conditions in Fennoscandia, in the immediate vicinity of a radiation-hazardous facility. This study comprehensively examined all dominant vegetation species across multiple layers of the boreal forest simultaneously. The research area included the territory surrounding the Kola NPP within a 15 km radius. Moreover, after obtaining a special permit, sampling was conducted within the nuclear plant’s sanitary protection zone.

As expected, ^137^Cs in the boreal forests of the Kola Peninsula exhibited high mobility. Our previous studies confirmed this by analyzing its modes of occurrence, revealing that up to 33% of the cesium introduced into soil samples transitioned into water-soluble and exchangeable forms, which are readily available for plant uptake [13]. Gamma-spectrometric measurements further demonstrated that the specific activity of ^137^Cs in all native plant parts was higher than in the underlying soils.

In a previous study of local soils, we assessed the area surrounding the Kola NPP as a territory with a relatively safe environment, in accordance with the adopted radioecological environmental standards for soils in the Russian Federation. However, assessing this for the studied plants is more challenging, as they are not edible and do not represent agricultural products, meaning no established regulations are available for them. Nevertheless, certain species, such as *Vaccinium myrtillus* and *Ledum palustre*, are recognized as potential medicinal raw materials and are included in the corresponding register [22]. For such plants, a specific activity threshold for ^137^Cs of no more than 400 Bq/kg dry weight has been established. The specific activities measured in all selected plant samples were below this limit, indicating that plant raw materials from local ecosystems do not pose a radiation hazard and can be safely used for medicinal purposes.

To assess differences in the intensity of ^137^Cs accumulation among plant species, specific activities in plant parts were compared as independent variables by using the *t*-test (Supplementary S5-2). Within-plant comparisons were conducted by using the *t*-test for dependent variables (Supplementary S5-1). When sample distributions deviated from normality, the Mann–Whitney U test was applied. If no significant differences were detected, transfer factors (*TF*) and above-ground transfer factors (*TFag*) were additionally compared across plant parts.

As a result, we found that the intensity of ^137^Cs accumulation in plant parts within northern taiga ecosystems followed the increasing trend shown below (where “<“ denotes a significant difference, while “≤” indicates the absence of a significant difference; in cases where no significant differences were found, parts are grouped within parentheses, and their order is determined based on mean values):

Birch branches < birch leaves ≤ (spruce needles ≤ spruce branches ≤ pine branches) < (pine needles ≤ rosemary branches) < bilberry branches < rosemary leaves < (Pleurozium ≤ blueberry branches ≤ bilberry leaves) < blueberry leaves.

Both European blueberry (*Vaccinium myrtillus*) and northern bilberry (*Vaccinium uliginosum*) demonstrated strong bioindicator properties for ^137^Cs. This could be attributed to their high physiological demand for potassium in nutrient-poor soils. Given the chemical similarity between ^137^Cs and potassium, these species actively absorbed cesium under conditions of potassium deficiency. Similar trends have been observed in other studies investigating ^137^Cs bioaccumulation in blueberries in both background and contaminated ecosystems [4,23,24].

We did not anticipate a significant impact of routine emissions from the Kola NPP on ^137^Cs uptake in vegetation. Neither proximity to the facility nor wind speed and frequency in the direction of the sampling sites resulted in elevated ^137^Cs activity in plant parts within the sanitary protection zone (SPZ) compared with the observation area and background site. On the contrary, in nearly all cases, ^137^Cs accumulation in plants was more pronounced at the background site.

This finding supports the hypothesis that soil properties governing ^137^Cs mobility in the soil–plant system may be more influential than the mere presence of a radiation-hazardous facility nearby. This finding supports the established views in the literature that soil properties governing ^137^Cs mobility in the soil–plant system play a pivotal role in its behavior [25]. Within the framework of our study, these properties proved to be more influential than the mere presence of a radiation-hazardous facility nearby.

### Correlation Analysis and Soil Influence on ^137^Cs Mobility

Correlation analysis confirmed this assumption, revealing significant relationships between the ^137^Cs, *TF*, and *TFag* in plants and the content of organic matter, mobile potassium, total potassium, forest litter storage, and exchangeable calcium and magnesium in soil. Spearman correlation coefficient (rₛ) values between soil properties and ^137^Cs accumulation factors in plant parts reached |0.93|, indicating a strong association.

Correlations between cesium bioaccumulation indices and soil properties such as physical clay and silt content were consistently negative. In these cases, the (rₛ) values were notably high but statistically significant only for *Ledum palustre*, which may be attributed to the limited sample size.

These findings support our hypothesis that under the nutrient-poor conditions of albic podzols, characterized by low organic matter content, nutrient depletion, and a lack of biophilic elements and clay minerals, ^137^Cs exhibits high mobility in soils and accumulates actively in vegetation. Similar conclusions were previously drawn by Finnish researchers, who artificially introduced radiocesium into soil and analyzed its uptake intensity under potassium-deficient podzolic soil conditions, as well as following the application of potassium fertilizers. Their findings demonstrated that fertilizer application reduced ^137^Cs uptake by pine trees by 5.2–43.0%, with higher fertilizer doses resulting in a stronger reduction and a faster plant response. With a single application, ^137^Cs content in conifers decreased by 13.5%, whereas repeated applications resulted in a 37.5% reduction compared with uptake from poorly fertilized soil [26,27,28]. The simultaneous application of ash and potassium chloride to sandy forest soils affected by Chernobyl contamination in the Drevlianskyi Natural Reserve in Ukraine reduced cesium uptake by plants by 45 percent [29]. Studies in beech forests in France and laboratory experiments on growing ryegrass also indicated potassium as the main competitor to cesium: with a decrease in the concentration of exchangeable potassium in the soil, the bioavailability of cesium for plants rises [30,31].

Our results confirmed a similar pattern, not only for trees but also for shrubs and mosses. The bioaccumulation coefficients (*TF* and *TFag*) of ^137^Cs in northern taiga vegetation exceeded the typical values observed in both background and contaminated landscapes of central Russia, including hydromorphic environments, and were comparable to those reported for northern taiga ecosystems in Scandinavian countries [4,25,32].

Thus, we conclude that ^137^Cs exhibits high mobility in the forests of the Kola Peninsula, posing a significant risk of transfer through food chains. Despite established concepts regarding cesium as a low-mobility radionuclide that is strongly sorbed by soil—a notion also supported by recent studies in Fukushima [33,34,35,36,37]—our results suggest that at high latitudes, cesium behavior may become considerably more mobile in northern taiga ecosystems.

## 5. Conclusions

In the northern taiga ecosystems of the area surrounding the Kola NPP, ^137^Cs demonstrated notable mobility within the soil–plant system, actively accumulating in the vegetation cover. The primary indicators of its bioaccumulation are plants of the Ericaceae family. No significant influence of the Kola NPP on cesium uptake by vegetation was detected.

Apparent ^137^Cs mobility appears to be primarily driven by soil properties—namely, the low content of nutrient elements and clay minerals—a conclusion supported by correlation analyses. On the Kola Peninsula, ^137^Cs mobility in the soil–plant system was considerably higher than in previously studied regions of Russia, Belarus, and Central European countries. This is likely attributable to the unique environmental conditions characteristic of Fennoscandia.

## Figures and Tables

**Table 1:** ^137^Cs content comparison in specific radioactivity of ^137^Cs.

^137^Cs Content	Observation Zone	Sanitary Protection Zone		Background	
Specific activity	**(n = 10)**	Bq/kg	*t*-Test	Bq/kg	*t*-Test
Branches	14.6	9.9	**2.94**	21.4	**−4.27**
Fir needles	19.9	13.3	**2.32**	26.9	**−2.3**

Note: Statistically significant differences, determined using Student’s *t*-test (*p* < 0.05; tₓᵣ = 2.26), are highlighted in bold.

**Table 2:** Spearman rank correlation coefficients between **^137^Cs** accumulation indicators in **Scots pine** and selected soil properties.

Index	K_2_O, mg/100 g	Ca^2+^ mmol/100 g	Mg^2+^ mmol/100 g	Litter Storage kg/m^2^	Organic Matter, kg/m^2^	K Storage, kg/m^2^
A_sp_ branches ^137^Cs, Bq/kg	−0.30	−0.42	−0.26	−0.29	−0.06	−0.57
A_sp_ fir needles, ^137^Cs, Bq/kg	−0.49	−0.55	−0.45	**−0.66**	−0.20	**−0.60**
TF branches	**−0.76**	**−0.87**	**−0.64**	**−0.59**	−0.55	**−0.71**
TF in fir needles	**−0.67**	**−0.80**	**−0.75**	**−0.75**	**−0.58**	**−0.65**
TF_ag_ in branches n·10^−3^ m^2^/kg	**−0.69**	−0.50	−0.38	−0.56	−0.40	**−0.83**
TF_ag_ in fir needles, n·10^−3^ m^2^/kg	**−0.74**	**−0.68**	**−0.75**	**−0.84**	**−0.53**	**−0.76**

Note 1: Values of rₛ indicating significant correlations are highlighted in bold. For n = 12 and *p* = 0.05, a value of |rₛ| > 0.58 is considered significant.

## Data Availability

Data is contained within the article or Supplementary Materials.

## Supplementary Materials

The following supporting information can be downloaded at: https://www.mdpi.com/article/10.3390/life15050774/s1. Supplementary File S1: Geobotanical Descriptions of Sites; Supplementary File S2: Specific activities of ^137^Cs and bioaccumulation indicators in the branches and needles; Supplementary File S3: Results of the comparison of average values of ^137^Cs contamination indicators in the observation zone; Supplementary File S4: Spearman’s rank correlation coefficients for ^137^Cs accumulation indicators in vegetation; Supplementary File S5: Comparison of the specific activities of ^137^Cs in the components of a single plant.

## Appendix A

Plants samples collected from the number of sites:

Tree layer: *Pinus sylvestris* (n = 12), *Picea abies* (n = 7), and *Betula pendula* (n = 12); understory layer: *Vaccinium mirtillus* (n = 12), *Vaccinium uliginosum* (n = 12), and *Ledum palustre* (n = 11); mosses: *Pleurozium schreberi* (n = 9).
