# Supplementary material for: Bioaccumulation of 137Cs: Vegetation Responses, Soil Interactions and Ecological Implications in the Northern Taiga Ecosystems
Source: Life (Basel). 2025 May 12;15(5):774. doi: 10.3390/life15050774 (PMC12113250; doi:10.3390/life15050774)
Supplement: Supplementary file 1 [file life-15-00774-s001.zip › Supplementary File S1.pdf]

Supplementary File S1.

Geobotanical Descriptions of Sites

The average age of the dominant trees on the sites (pine, spruce) ranged from 60 to 80 years.

Site S-1
06.08.14
The forest type formed in these growing conditions is characterized by the predominance of Scots pine (*Pinus sylvestris* L.), with the participation of other tree species—silver birch and pubescent birch (*Betula pendula* Roth. × *Betula pubescens* Ehrh) and Siberian spruce (*Picea obovata* Ledeb.). The vitality of the stand: no signs of weakening (Category 1 for coniferous and deciduous trees on the tree condition scale).
In the herbaceous and shrub layer, the predominant species are from the *Vaccinium* genus: lingonberry (*Vaccinium vitis-idaea* L.) and blueberry (*Vaccinium myrtillus* L.). There are occurrences of crowberry (*Empetrum hermaphroditum* Lange ex Hagerup), bog bilberry (*Vaccinium uliginosum* L.), and bog rosemary (*Ledum palustre* L.).
The lichen cover is very characteristic, consisting of the lichen *Cladonia stellaris* (Opiz) Pouzar & Wezda. On stones, there is a common mosaic of crustose epilithic lichens from the genera *Bellemerea*, *Lecidea*, and *Porpidia*.
Species composition: 10P.
Species status: Pine
Projected cover of blueberry: 100%.
Forest type: pine-blueberry-lichen forest (*Pinetum cladomyrtillosum boreale*, according to: Sambuk, 1932, *Pinetum myrtillosocladinosum*, according to: Avrorin et al., 1936, floristic classification syntaxon *Cladonio arbuscuiae-Pinetum sylvestris* subass. *vaccinium myrtilli*, according to: Morozova et al., 2008).

Site S-2
08.08.14
The forest type formed in these growing conditions is characterized by the predominance of Scots pine (*Pinus sylvestris* L.), with the participation of other tree species—silver birch and pubescent birch (*Betula pendula* Roth. × *Betula pubescens* Ehrh) and Siberian spruce (*Picea obovata* Ledeb.). The vitality of the stand: no signs of weakening (Category 1 for coniferous and deciduous trees on the tree condition scale).
In the herbaceous and shrub layer, Labrador tea (*Ledum palustre* L.) predominates, as well as blueberry (*Vaccinium myrtillus* L.) and bog bilberry (*Vaccinium uliginosum* L.).
In the moss-lichen layer, green mosses *Pleurozium schereberi* (Brid) Mitt. and *Hylocomium splendens* Hedw. dominate.
Species composition: 10P.
Species status: Pine
Projected cover of blueberry: 100%.
Forest type: pine-Labrador tea-blueberry-green moss forest, according to: Martynenko, 1999 (*Pinetum ledosomyrtillosum*, according to: Rysin, Savelieva, 2008).

Site P-1
14.08.14
The forest type formed in these growing conditions is characterized by the predominance of

Scots pine (*Pinus sylvestris* L.). The vitality of the stand: no signs of weakening (Category 1 for coniferous trees on the tree condition scale).
In the herbaceous and shrub layer, blueberry (*Vaccinium myrtillus* L.) and lingonberry (*Vaccinium vitis-idaea* L.) dominate, with occurrences of crowberry (*Empetrum hermaphroditum* Lange ex Hagerup).
The lichen cover is very characteristic, consisting of the lichen *Cladonia stellaris* (Opiz) Pouzar & Wezda.
Species composition: 10P.
Species status: Pine
Projected cover of blueberry: 50%.
Forest type: pine-blueberry-lichen forest (*Pinetum cladomyrtillosum boreale*, according to: Sambuk, 1932, *Pinetum myrtillosocladinosum*, according to: Avrorin et al., 1936, floristic classification syntaxon *Cladonio arbuscuiae-Pinetum sylvestris* subass. *vaccinium myrtilli*, according to: Morozova et al., 2008).

Site P-2
10.08.14
The forest type formed in these growing conditions is characterized by the predominance of Scots pine (*Pinus sylvestris* L.), with the participation of other tree species—silver birch and pubescent birch (*Betula pendula* Roth. × *Betula pubescens* Ehrh), willows (*Salix caprea* L. and *Salix hastata* L.), and common juniper (*Juniperus communis* L.). The vitality of the stand: no signs of weakening (Category 1 for coniferous and deciduous trees on the tree condition scale).
In the herbaceous and shrub layer, blueberry (*Vaccinium myrtillus* L.) predominates. There are occurrences of crowberry (*Empetrum hermaphroditum* Lange ex Hagerup), lingonberry (*Vaccinium vitis-idaea* L.), bog bilberry (*Vaccinium uliginosum* L.), and Labrador tea (*Ledum palustre* L.).
In the moss-lichen layer, the dominant lichens are *Cladonia stellaris* (Opiz) Pouzar & Wezda and *Cladonia rangiferina* (L.) Weber ex F.N. Wigg. Lichens such as *Cladonia deformis* (L.) Hoffm. and *Cetraria islandica* (L.) Ach. are also present. Green mosses *Pleurozium schereberi* (Brid) Mitt. and *Hylocomium splendens* Hedw. predominate.
Species composition: 8P2S+B.
Species status: Pine, spruce with birch inclusions
Projected cover of blueberry: 100%.
Forest type: pine-green moss-lichen blueberry forest (*Pinetum hylocomiosocladinosum*, floristic classification syntaxon *Flavocerario nivalis-Pinetum sylvestris* subass. *typicum*, according to: Neshataev, Neshataeva, 2002).

Site P-3
14.08.14
The forest type formed in these growing conditions is characterized by the predominance of Scots pine (*Pinus sylvestris* L.), with the inclusion of silver birch and pubescent birch (*Betula pendula* Roth. × *Betula pubescens* Ehrh). The vitality of the stand: no signs of weakening (Category 1 for coniferous and deciduous trees on the tree condition scale).
In the herbaceous and shrub layer, blueberry (*Vaccinium myrtillus* L.) predominates. There are occurrences of Labrador tea (*Ledum palustre* L.).
In the moss-lichen layer, the dominant green mosses are *Pleurozium schereberi* (Brid) Mitt. and

Hylocomium splendens Hedw., with lichens *Cladonia rangiferina* (L.) Weber ex. F.N. Wigg and *Cladonia stellaris* (Opiz) Pouzar & Wezda.
Species composition: 8P2S+B.
Species status: Pine, spruce with birch inclusions
Projected cover of blueberry: 100%.
Forest type: pine-green moss-lichen blueberry forest (*Pinetum hylocomiosocladinosum*, floristic classification syntaxon *Flavocerario nivalis-Pinetum sylvestris* subass. *typicum*, according to: Neshataev, Neshataeva, 2002).

Site P-4
08.08.14
The forest type formed in these growing conditions is characterized by the predominance of Siberian spruce (*Picea obovata* Ledeb.), with the participation of other tree species—Scots pine (*Pinus sylvestris* L.) and silver birch and pubescent birch (*Betula pendula* Roth. × *Betula pubescens* Ehrh). The vitality of the stand: no signs of weakening (Category 1 for coniferous and deciduous trees on the tree condition scale).
In the herbaceous and shrub layer, blueberry (*Vaccinium myrtillus* L.) predominates, with occurrences of lingonberry (*Vaccinium vitis-idaea* L.), bog bilberry (*Vaccinium uliginosum* L.), crowberry (*Empetrum hermaphroditum* Lange ex Hagerup), and Labrador tea (*Ledum palustre* L.).
In the moss-lichen layer, the dominant green mosses are *Pleurozium schereberi* (Brid) Mitt. and *Hylocomium splendens* Hedw., with lichens such as *Cladonia stellaris* (Opiz) Pouzar & Wezda and *Cladonia rangiferina* (L.) Weber ex. F.N. Wigg.
Species composition: 7S3P+B.
Species status: Spruce, pine with birch inclusions.
Projected cover of blueberry: 75%.
Forest type: spruce-blueberry-green moss forest (*Eu-Piceetum myrtilletosum*, according to: Morozova, Korotkov, 1999; *Piceetum myrtillosum*, according to: Rysin, Savelieva, 2002; floristic classification syntaxon *Linnaeo borealis-Piceetum abietis* subass. *myrtilletosum* var. *typica*).

Site P-5
12.08.14
The forest type formed in these growing conditions is characterized by the predominance of Scots pine (*Pinus sylvestris* L.), with the participation of other tree species—silver birch and pubescent birch (*Betula pendula* Roth. × *Betula pubescens* Ehrh), Siberian spruce (*Picea obovata* Ledeb.), and common juniper (*Juniperus communis* L.). The vitality of the stand: no signs of weakening (Category 1 for coniferous and deciduous trees on the tree condition scale).
In the herbaceous and shrub layer, blueberry (*Vaccinium myrtillus* L.) predominates. Lingonberry (*Vaccinium vitis-idaea* L.), crowberry (*Empetrum hermaphroditum* Lange ex Hagerup), bog bilberry (*Vaccinium uliginosum* L.), and Labrador tea (*Ledum palustre* L.) are also found.
In the moss-lichen layer, lichens dominate, with *Cladonia stellaris* (Opiz) Pouzar & Wezda and *Cladonia rangiferina* (L.) Weber ex. F.N. Wigg. Green mosses are predominantly *Pleurozium schereberi* (Brid) Mitt. and *Hylocomium splendens* Hedw.
Species composition: 8P2S+B.

Species status: Pine, spruce with birch inclusions.
Projected cover of blueberry: 100%.
Forest type: green moss-lichen blueberry pine forest (*Pinetum hylocomiosocladinosum*, blueberry pine forest, floristic classification syntaxon *Flavocerario nivalis-Pinetum sylvestris* subass. *typicum*, according to: Neshataev, Neshataeva, 2002).

Site C-I
07.08.14
The forest type formed in these growing conditions is characterized by the predominance of Scots pine (*Pinus sylvestris* L.), with the admixture of silver birch and pubescent birch (*Betula pendula* Roth. × *Betula pubescens* Ehrh.). The vitality of the stand: no signs of weakening (Category 1 for coniferous and deciduous trees on the tree condition scale).
In the herbaceous and shrub layer, blueberry (*Vaccinium myrtillus* L.) predominates. Lingonberry (*Vaccinium vitis-idaea* L.), crowberry (*Empetrum hermaphroditum* Lange ex Hagerup), and bog bilberry (*Vaccinium uliginosum* L.) are also found.
The lichen cover is very characteristic and consists of the lichen *Cladonia stellaris* (Opiz) Pouzar & Wezda. On stones, a mosaic of crustose epilithic lichens of the genera *Bellemerea*, *Lecidea*, and *Porpidia* is common.
Species composition: 10P.
Species status: Pine.
Projected cover of blueberry: 63%.
Forest type: blueberry-lichen pine forest (*Pinetum cladomyrtillosum boreale*, according to: Sambuk, 1932, *Pinetum myrtillosocladinosum*, according to: Avrorin et al., 1936, floristic classification syntaxon *Cladonio arbuscuiae-Pinetum sylvestris* subass. *vaccinium myrtilli*, according to: Morozova et al., 2008).

Site C-II
20.08.14
The forest type formed in these growing conditions is characterized by the predominance of Scots pine (*Pinus sylvestris* L.), with the participation of other tree species such as Siberian spruce (*Picea obovata* Ledeb.) and silver and pubescent birch (*Betula pendula* Roth. × *Betula pubescens* Ehrh.). The vitality of the stand: no signs of weakening (Category 1 for coniferous and deciduous trees on the tree condition scale).
In the herbaceous and shrub layer, blueberry (*Vaccinium myrtillus* L.) and crowberry (*Empetrum hermaphroditum* Lange ex Hagerup) dominate. Lingonberry (*Vaccinium vitis-idaea* L.), bog bilberry (*Vaccinium uliginosum* L.), and bog rosemary (*Ledum palustre* L.) are also present.

In the moss and lichen cover, the dominant species include the lichen *Cladonia stellaris* (Opiz) Pouzar & Wezda, *Cadonia rangiferina* (L.) Weber ex F.N. Wigg, and *Cladonia deformis* (L.) Hoffm. The lichen *Cetraria islandica* (L.) Ach. is also found. Among the green mosses, *Polytrichum commune* Hedw. is common. On rocks, a mosaic of crustose epilithic lichens of the genera *Bellemerea*, *Lecidea*, and *Porpidia* is typical.
Species composition: 10P.
Species status: Pine.
Projected cover of blueberry: 88%.
Forest type: blueberry-lichen pine forest (*Pinetum cladomyrtillosum boreale*, according to: Sambuk, 1932, *Pinetum myrtillosocladinosum*, according to: Avrorin et al., 1936, floristic

classification syntaxon *Cladonio arbuscuiae-Pinetum sylvestris* subass. *vaccinium myrtilli*, according to: Morozova et al., 2008).

Site C-III
18.08.14
The forest type formed in these growing conditions is characterized by the predominance of Scots pine (*Pinus sylvestris* L.), with the participation of other tree species such as silver and pubescent birch (*Betula pendula* Roth. × *Betula pubescens* Ehrh.), Siberian spruce (*Picea obovata* Ledeb.), goat willow (*Salix caprea* L.), and rowan (*Sorbus gorodkovii* Pojark.). The vitality of the stand: no signs of weakening (Category 1 for coniferous and deciduous trees on the tree condition scale).
In the herbaceous and shrub layer, blueberry (*Vaccinium myrtillus* L.) dominates. Lingonberry (*Vaccinium vitis-idaea* L.), crowberry (*Empetrum hermaphroditum* Lange ex Hagerup), bog bilberry (*Vaccinium uliginosum* L.), and bog rosemary (*Ledum palustre* L.) are also present.

In the moss and lichen cover, the dominant species is the lichen *Cladonia stellaris* (Opiz) Pouzar & Wezda. Among the green mosses, *Pleurozium schereberi* (Brid) Mitt. and *Hylocomium splendens* Hedw. prevail.
Species composition: 10P.
Species status: Pine.
Projected cover of blueberry: 75%.
Forest type: green moss-lichen blueberry pine forest (*Pinetum hylocomiosocladinosum*, floristic classification syntaxon *Flavocerario nivalis-Pinetum sylvestris* subass. *typicum*, according to: Neshataev, Neshataeva, 2002).

Site C-IV
18.08.14
The forest type formed in these growing conditions is characterized by the predominance of Scots pine (*Pinus sylvestris* L.), with the participation of other tree species such as silver and pubescent birch (*Betula pendula* Roth. × *Betula pubescens* Ehrh.) and Siberian spruce (*Picea obovata* Ledeb.). The vitality of the stand: no signs of weakening (Category 1 for coniferous and deciduous trees on the tree condition scale).
In the herbaceous and shrub layer, blueberry (*Vaccinium myrtillus* L.) dominates. Lingonberry (*Vaccinium vitis-idaea* L.) is also present.

In the moss and lichen cover, the dominant lichen is *Cadonia rangiferina* (L.) Weber ex. F.N. Wigg. *Cladonia stellaris* (Opiz) Pouzar & Wezda is also present. Among the green mosses, *Pleurozium schereberi* (Brid) Mitt. and *Hylocomium splendens* Hedw. predominate.
Species composition: 7P3S.
Species status: Pine, spruce.
Projected cover of blueberry: 75%.
Forest type: green moss-lichen blueberry pine forest (*Pinetum hylocomiosocladinosum*, floristic classification syntaxon *Flavocerario nivalis-Pinetum sylvestris* subass. *typicum*, according to: Neshataev, Neshataeva, 2002).

Site B
13.08.14
The forest type formed in these growing conditions is characterized by the predominance of

Scots pine (*Pinus sylvestris* L.). The vitality of the stand: no signs of weakening (Category 1 for coniferous and deciduous trees on the tree condition scale).
In the herbaceous and shrub layer, blueberry (*Vaccinium myrtillus* L.) dominates. Crowberry (*Empetrum hermaphroditum* Lange ex Hagerup) and lingonberry (*Vaccinium vitis-idaea* L.) are also present.

The lichen cover is very characteristic, consisting of the lichen *Cladonia stellaris* (Opiz) Pouzar & Wezda. On the branches of trees, epiphytic lichens of the genera *Bryoria* and *Hypogymnia* are common.
Species composition: 10P.
Species status: Pine.
Projected cover of blueberry: 63%.
Forest type: blueberry-lichen pine forest (*Pinetum cladomyrtillosum boreale*, according to: Sambuk, 1932, *Pinetum myrtillosocladinosum*, according to: Avrorin et al., 1936, floristic classification syntaxon *Cladonio arbuscuiae-Pinetum sylvestris* subass. *vaccinium myrtilli*, according to: Morozova et al., 2008).

References:

1) Morozova O.V., Zaugolnova L.B., Isaeva L.G., Kostina V.A. Classification of Boreal Forests in the North of European Russia. I. Oligotrophic Coniferous Forests // Vegetation of Russia. 2008. №13. P.61-81
2) Avrorin N.A., Kachurin M.Kh., Korovkin A.A. Materials on the vegetation of the Khibiny Mountains. Materials on the vegetation of the central and western parts of the Kola Peninsula // Proceedings of the Council for the Study of Productive Forces. Series Kola. Vol. 11. 1936. Pp. 3-93.
3) Sambuk F.V. The forests of the Pechora region // Transactions of the Botanical Museum of the USSR Academy of Sciences. 1932. Vol. 24. Pp. 63-250.
4) Rysin L.P., Savelieva L.I. Pine forests of Russia. Moscow: Scientific Publishing Society "KMK", 2008. 289 p.
5) Morozova O.V., Korotkov V.N. Classification of the forest vegetation of the Kostomuksha Nature Reserve // Protected Area Studies. Issue 5. Pp. 56-78.
6) Neshataev V.Yu., Neshataeva V.Yu. Syntaxonomic diversity of pine forests in the Lapland Nature Reserve // Botanical Journal. 2002. Vol. 87. No. 1. Pp. 99–106.
7) Martynenko, V.B. Syntaxonomic analysis of the forests of the Bashkir State Nature Reserve: Specialty 03.00.05: Abstract of the dissertation for the degree of Candidate of Biological Sciences / Martynenko Vasily Borisovich. – Ufa, 1999. – 16 p.
