# Supplementary material for: Bioaccumulation of 137Cs: Vegetation Responses, Soil Interactions and Ecological Implications in the Northern Taiga Ecosystems
Source: Life (Basel). 2025 May 12;15(5):774. doi: 10.3390/life15050774 (PMC12113250; doi:10.3390/life15050774)
Supplement: Supplementary file 1 [file life-15-00774-s001.zip › Supplementary File S2.pdf]

Supplementary File S2

Supplementary S2-1. Specific Activities of ^{137}Cs and Bioaccumulation Indicators in the Branches and Needles of Scots Pine.

Sampling Site	Branches			Needles		
	Specific Activity, Bq/kg	TFag, $n \times 10^{-3}$ m^2/kg	TF	Specific Activity, Bq/kg	TFag, $n \times 10^{-3}$ m^2/kg	TF
S-1	9,9±5,6	13±9	3,90±2,36	13,3±2,5	18±8	5,28±1,53
S-2	15,4±3,7	15±5	3,82±1,24	7,2±2,1	7±2	1,79±0,65
P-1	19,3±3,4	15±4	4,43±1,32	23,4±3,1	18±5	5,38±1,47
P-2	12,0±2,4	5±1	1,72±0,40	9,2±2,9	4±1	1,31±0,44
P-3	14,4±3,7	18±7	7,68±3,22	19,1±4,6	24±10	10,21±4,17
P-4	10,9±2,7	11±3	2,87±0,93	22,9±3,0	22±6	6,02±1,51
P-5	10,7±3,8	11±5	4,18±1,74	18,1±3,2	19±5	7,05±2,02
C-I	26,2±3,5	49±23	17,08±8,04	34,5±3,7	65±30	22,48±10,44
C-II	12,7±4,1	7±2	1,94±0,67	21,4±3,2	11±2	3,26±0,65
C-III	9,0±2,6	10±4	3,96±1,61	11,5±2,4	12±4	5,04±1,78
C-IV	15,3±3,2	10±3	5,20±1,38	31,6±4,0	22±5	10,75±2,25
B	21,4±3,0	21±6	7,94±2,28	26,9±3,8	26±8	10,01±2,89

Supplementary S2-2. Specific activities of ^{137}Cs and bioaccumulation indicators in the branches and needles of Norway spruce.

Sampling Site	Branches			Needles		
	Specific Activity, Bq/kg	TFag, $n \times 10^{-3}$ m^2/kg	TF	Specific Activity, Bq/kg	TFag, $n \times 10^{-3}$ m^2/kg	TF
P-1	13,2±2,9	10±3	3,19±0,71	9,7±2,0	8±2	2,33±0,49
P-4	6,7±2,1	7±3	1,77±0,56	12,1±2,1	12±3	3,19±0,56
P-5	15,1±3,0	16±5	5,94±1,20	9,0±1,7	9±3	3,55±0,68
C-II	13,3±2,3	7±2	2,05±0,36	13,4±2,3	7±2	2,06±0,36
C-III	9,3±2,0	10±4	4,13±0,90	7,6±1,7	8±3	3,37±0,76
C-IV	10,5±3,6	7±3	3,13±1,06	15,6±2,7	11±3	4,65±0,82
B	15,2±3,2	15±5	5,70±1,18	14,8±2,5	14±4	5,54±0,95

Supplementary S2-3. Specific activities of ^{137}Cs and bioaccumulation indicators in the branches and leaves of silver birch.

Sampling Site	Branches			Needles		
	Specific Activity, Bq/kg	TFag, $n \times 10^{-3}$ m^2/kg	TF	Specific Activity, Bq/kg	TFag, $n \times 10^{-3}$ m^2/kg	TF
S-1	6,1±1,7	10±5	2,42±0,69	13,2±3,6	21±11	5,24±1,41
S-2	7,5±2,4	7±3	1,88±0,61	<2,5	-	-
P-1	<2,5	-*	-	18,6±5,2	15±5	4,49±1,25

Sampling Site	Branches			Needles		
	Specific Activity, Bq/kg	TFag, n×10^{-3} m^2/kg	TF	Specific Activity, Bq/kg	TFag, n×10^{-3} m^2/kg	TF
P-2	<2,5	-	-	15,8±3,5	6±2	2,27±0,50
P-3	8,3±2,2	10±4	4,48±1,20	7,4±2,4	9±4	3,98±1,31
P-4	6,0±1,8	6±2	1,58±0,48	14,7±3,6	14±5	3,89±0,94
P-5	<2,5	-	-	14,0±3,3	15±5	5,50±1,27
C-I	9,9±3,1	19±10	6,46±2,00	17,9±4,2	34±7	11,72±2,73
C-II	5,9±2,5	3±1	0,91±0,39	10,6±3,5	6±2	1,62±0,53
C-III	4,7±2,0	5±3	2,07±0,90	15,4±4,3	17±7	6,82±1,89
C-IV	10,3±3,1	7±2	3,07±0,92	13,3±4,0	9±3	3,96±1,18
B	8,1±2,2	8±3	3,04±0,84	17,5±4,5	17±6	6,56±1,67

Supplementary S2-4. Specific activities of ^{137}Cs and bioaccumulation indicators in the branches and leaves of European blueberry.

Sampling Site	Branches			Leaves		
	Specific Activity, Bq/kg	TFag, n×10^{-3} m^2/kg	TF	Specific Activity, Bq/kg	TFag, n×10^{-3} m^2/kg	TF
S-1	34,9±6,5	46±20	13,83±3,95	13,0±10,9	17±16	5,14±4,47
S-2	50,0±5,9	48±12	12,44±3,11	61,4±9,6	59±16	15,27±4,14
P-1	87,4±6,9	68±17	20,10±5,05	164,8±17,1	129±34	37,89±9,87
P-2	38,1±7,6	16±4	5,46±1,26	58,0±12,5	24±6	8,30±2,04
P-3	35,8±5,1	44±16	19,11±6,88	71,7±13,4	88±34	38,27±14,52
P-4	37,8±4,7	37±9	9,93±2,46	50,8±8,3	49±13	13,36±3,59
P-5	25,9±4,9	27±8	10,11±2,97	56,4±10,2	58±17	22,00±6,33
C-I	80,2±7,2	51±70	52,22±24,05	115,5±13,9	218±102	75,16±35,15
C-II	31,0±8,2	17±5	4,72±1,39	41,5±13,6	22±8	6,33±2,24
C-III	68,6±8,1	74±23	30,06±9,37	70,9±12,6	77±26	31,08±10,52
C-IV	59,8±6,6	41±8	20,37±4,08	65,7±11,1	45±11	22,37±5,31
B	90,3±8,7	87±23	33,56±9,03	120,4±15,6	117±33	44,77±12,66

Supplementary S2-5. Specific activities of ^{137}Cs and bioaccumulation indicators in the branches of northern bilberry.

Sampling Site	Branches			Leaves		
	Specific Activity, Bq/kg	TFag, n×10^{-3} m^2/kg	TF	Specific Activity, Bq/kg	TFag, n×10^{-3} m^2/kg	TF
S-1	67,0±32,2	105±56	26,56±12,76	60,0±30,6	94±52	23,79±12,12
S-2	19,0±3,7	18±5	4,76±0,94	37,2±7,4	36±11	9,31±1,85
P-1	27,4±8,1	22±8	6,62±1,95	54,7±18,5	43±18	13,22±4,47
P-2	14,0±3,0	6±1	2,01±0,44	45,4±11,8	19±5	6,54±1,70
P-3	25,8±5,3	32±12	13,85±2,82	66,0±16,7	82±34	35,46±8,96
P-4	17,6±3,9	17±5	4,64±1,04	45,0±8,5	44±12	11,88±2,24

Sampling Site	Branches			Leaves		
	Specific Activity, Bq/kg	TFag, $n\times10^{-3}$ m^2/kg	TF	Specific Activity, Bq/kg	TFag, $n\times10^{-3}$ m^2/kg	TF
P-5	50,5±35,0	52±38	19,78±13,71	79,0±41,1	82±46	30,96±16,09
C-I	32,2±8,8	61±32	21,07±5,76	59,6±29,9	113±76	39,02±19,61
C-II	30,0±7,2	16±4	4,62±1,10	49,2±12,5	26±8	7,57±1,93
C-III	11,9±2,9	13±5	5,27±1,30	25,8±6,3	28±11	11,39±2,77
C-IV	26,1±4,8	18±4	7,75±1,44	67,5±22,7	47±18	20,09±6,77
B	20,1±3,4	20±6	7,53±1,29	27,0±6,1	26±9	10,11±2,26

Supplementary S2-6. Specific activities of ^{137}Cs and bioaccumulation indicators in the branches and leaves of Bog rosemary.

Sampling Site	Branches			Leaves		
	Specific Activity, Bq/kg	TFag, $n\times10^{-3}$ m^2/kg	TF	Specific Activity, Bq/kg	TFag, $n\times10^{-3}$ m^2/kg	TF
S-1	39,9±9,1	63±20	15,81±16,45	58,3±10,5	92±26	23,10±19,03
S-2	27,7±4,5	27±7	6,92±5,07	47,2±8,8	46±13	11,80±10,02
P-1	23,8±4,7	19±6	5,75±4,73	72,5±12,7	57±17	17,51±12,86
P-2	18,4±3,6	8±2	2,65±4,33	35,5±6,8	15±3	5,11±8,28
P-3	21,9±3,9	27±10	11,73±6,28	65,6±11,7	81±31	35,20±19,01
P-4	12,1±2,5	12±4	3,19±3,14	38,67,4	38±11	10,19±9,16
P-5	31,8±5,3	33±9	12,47±9,25	94,6±17,4	98±28	37,09±30,42
C-I	19,2±4,5	36±19	12,58±6,49	46,8±8,6	89±43	30,63±12,54
C-II	20,9±4,0	11±3	3,22±4,67	37,3±7,7	20±5	5,73±9,10
C-III	8,4±2,0	9±3	3,73±3,11	12,6±5,1	14±7	5,57±7,78
B	15,9±3,0	15±5	5,93±4,54	30,9±6,1	30±10	11,57±9,06

Supplementary S2-7. Specific activities of ^{137}Cs and bioaccumulation indicators in the mat of Pleurozium.

Sampling Site	Specific Activity, Bq/kg	TFag, $n\times10^{-3}$ m^2/kg	TF
S-2	54,73±11,35	53±11	13,68±2,84
P-1	55,52±11,45	44±9	13,41±2,77
P-2	43,17±9,32	18±4	6,22±1,34
P-3	35,86±7,70	44±10	19,26±4,13
P-4	48,44±16,22	47±16	12,78±4,28
P-5	34,04±7,80	35±8	13,34±3,06
C-II	14,99±4,96	8±3	2,30±0,76
C-III	27,24±7,39	30±8	12,04±3,27
C-IV	94,47±30,07	65±21	28,10±8,94
B	85,30±17,02	83±17	31,90±6,36
