# Supplementary material for: Bioaccumulation of 137Cs: Vegetation Responses, Soil Interactions and Ecological Implications in the Northern Taiga Ecosystems
Source: Life (Basel). 2025 May 12;15(5):774. doi: 10.3390/life15050774 (PMC12113250; doi:10.3390/life15050774)
Supplement: Supplementary file 1 [file life-15-00774-s001.zip › Supplementary File S3.pdf]

Supplementary File S3

Supplementary S3-1. Results of the comparison of average values of ^{137}Cs contamination indicators in the observation zone (OZ) and the background site (Norway spruce).

Indicator	Average for OZ (n=10)	Background Site	
		Value	Observed t-statistic
Asp of ^{137}Cs in branches, Bq/kg	11,4	15,2	**-3,0**
Asp of ^{137}Cs in needles, Bq/kg	11,2	14,8	**-2,9**
TN in branches	3,4	5,70	**-3,8**
TN in needles	3,2	5,54	**-6,2**
TFag in branches, n×10^{-3} m^2/kg	0,010	0,015	**-3,9**
TFag in needles, n×10^{-3} m^2/kg	0,009	0,014	**-6,5**

Note: Significant differences are highlighted in bold when comparing using the Student's t-test ($p<0.05$; tкр. = 2.26).

Supplementary S3-2. Results of comparison of average values of 137Cs contamination indicators in the sampling area near the Kola nuclear power plant (Kola NPP) and the background site (Silver birch).

Indicator	Average for OZ (n=10)	SPZ of Kola NPP		Background Site	
		Value	Observed t-statistic	Value	Observed t-statistic
Asp of ^{137}Cs in branches, Bq/kg	7,5	6,1	-0,7	8,1	-2,2
Asp of ^{137}Cs in leaves, Bq/kg	14,2	13,2	-0,2	17,5	**-2,7**
TN in branches	2,9	2,42	0,7	3,04	-0,2
TN in leaves	4,9	5,24	-0,3	6,56	-1,7
TFag in branches, n×10^{-3} m^2/kg	0,008	0,010	-0,9	0,008	0,1

Note: Significant differences are highlighted in bold when comparing using the Student's t-test ($p<0.05$; tкр. = 2.26).

Supplementary S3-3. Results of comparison of average values of 137Cs contamination indicators in the sampling area near the Kola Nuclear Power Plant (Kola NPP) and the background site (European blueberry).

Indicator	Average for OZ (n=10)	SPZ of Kola NPP		Background Site	
		Value	Observed t-statistic	Value	Value
Asp of ^{137}Cs in branches, Bq/kg	51,5	34,9	**2,5***	90,3	**-5,7**
TN in leaves	27,0	5,1	**3,4**	44,8	**-2,8**

Note: Significant differences are highlighted in bold when comparing using the Student's t-test ($p<0.05$; tкр. = 2.26).

Supplementary S3-4. Results of comparison of average values of 137Cs contamination indicators in the sampling area near the Kola Nuclear Power Plant (Kola NPP) and the background site (Northern bilberry).

Indicator	Average for OZ (n=10)	SPZ of NPP		Background Site	
		Value	Observed t-statistic	Value	Observed t-statistic
Asp of ^{137}Cs in branches, Bq/kg	25,5	67,0	**-11,8**	20,1	1,5
Asp of ^{137}Cs in leaves, Bq/kg	52,9	60	-1,4	27	**5,2**

Note: Significant differences are highlighted in bold when comparing using the Student's t-test (p<0.05; tкр. = 2.26).

Supplementary S3-5. Results of comparison of average values of 137Cs contamination indicators in the sampling area near the Kola Nuclear Power Plant (Kola NPP) and the background site (Bog rosemary).

Indicator	Average for OZ (n=10)	SPZ of Kola NPP		Background Site	
		Value	Observed t-statistic	Value	Observed t-statistic
Asp of ^{137}Cs in branches, Bq/kg	20,5	39,9	**-8,1**	15,9	1,9
Asp of ^{137}Cs in leaves, Bq/kg	50,1	58,3	-1,0	30,9	**2,4**
TN in leaves	17,6	23,10	-1,2	11,57	1,4
TFag in branches, n×10^{-3} m^2/kg	0,020	0,063	**-11,8**	0,015	1,4
TFag in leaves, n×10^{-3} m^2/kg	0,051	0,092	**-3,8**	0,030	1,9

Note: Significant differences are highlighted in bold when comparing using the Student's t-test (p<0.05; tкр. = 2.26).

Supplementary S3-6. Specific activities of ^{137}Cs and bioaccumulation indicators in the Pleurozium schreberi mat.

Sampling Site	Specific Activity, Bq/kg	TFag, n×10^{-3} m^2/kg	TF
S-2	54,73±11,35	53±11	13,68±2,84
P-1	55,52±11,45	44±9	13,41±2,77
P-2	43,17±9,32	18±4	6,22±1,34
P-3	35,86±7,70	44±10	19,26±4,13
P-4	48,44±16,22	47±16	12,78±4,28
P-5	34,04±7,80	35±8	13,34±3,06
C-II	14,99±4,96	8±3	2,30±0,76
C-III	27,24±7,39	30±8	12,04±3,27
C-IV	94,47±30,07	65±21	28,10±8,94
B	85,30±17,02	83±17	31,90±6,36
