# Supplementary material for: Bioaccumulation of 137Cs: Vegetation Responses, Soil Interactions and Ecological Implications in the Northern Taiga Ecosystems
Source: Life (Basel). 2025 May 12;15(5):774. doi: 10.3390/life15050774 (PMC12113250; doi:10.3390/life15050774)
Supplement: Supplementary file 1 [file life-15-00774-s001.zip › Supplementary File S4.pdf]

Supplementary File S4

Spearman's rank correlation coefficients for ^{137}Cs accumulation indicators in vegetation.

For Specific Activities

	K_2O, mg/100g	Ca^{2+}, mmol+/100g	Mg^{2+}, mmol+/100r	<0,001, %	<0,01, %	H, mmol+	Litter stock, kg/m²	Humus stock, kg/m²	Potassium stock, kg/m^2	Distance from the NPP, km	Wind recurrence, %	Wind speed, m/s	Elevation above sea level, m	pH H_2O, horizon O	pH H_2O, horizon AO	pH KCl, horizon O	pH KCl, horizon AO
Pine branches	-0,30	-0,42	-0,26	-0,39	-0,04	-0,12	-0,29	0,06	-0,57	0,34	-0,04	0,29	-0,13	0,21	-0,01	-0,14	0,15
Pine needles	-0,49	-0,55	-0,45	-0,31	0,15	-0,27	**-0,66***	-0,20	**-0,60**	0,56	0,14	-0,29	-0,30	-0,25	0,01	0,17	0,26
Blueberry branches	-0,29	-0,33	-0,06	-0,09	0,04	0,01	-0,03	0,09	-0,47	**0,66**	0,35	0,23	0,29	-0,02	-0,17	-0,44	-0,13
Blueberry leaves	-0,50	-0,49	-0,24	-0,14	-0,07	-0,17	-0,07	-0,03	**-0,59**	0,54	0,36	0,06	0,06	-0,01	-0,07	-0,46	-0,14
Bilberry branches	-0,37	-0,45	-0,20	**-0,63**	-0,43	**-0,73**	-0,52	-0,49	-0,26	-0,25	-0,43	-0,02	-0,37	0,36	0,45	0,55	**0,67**

**For Specific Activities**																	
Bilberry leaves	-0,32	-0,52	-0,41	-0,44	-0,22	-0,55	-0,31	-0,53	-0,15	-0,37	-0,25	-0,28	-0,45	0,13	0,42	**0,58**	0,39
Pleurozium Schreber	-0,41	-0,48	-0,15	-0,18	0,13	-0,19	-0,28	-0,10	**-0,60**	0,16	0,17	0,13	-0,07	0,37	0,08	-0,03	0,05
Bog rosemary branches	-0,11	-0,25	0,11	-0,54	**-0,65**	-0,55	-0,06	-0,25	-0,04	**-0,73**	-0,40	0,28	-0,45	**0,72**	0,37	0,26	0,39
Bog rosemary leaves	-0,29	-0,29	0,00	-0,40	**-0,75**	-0,45	-0,19	-0,25	-0,30	**-0,65**	-0,21	-0,01	**-0,64**	0,51	0,38	0,21	0,14
Spruce branches	-0,54	-0,50	-0,32	-0,43	0,11	**-0,86**	-0,54	-0,39	-0,29	0,14	-0,47	0,16	0,11	**0,61**	0,39	0,11	**0,68**

**For Specific Activities**																	
Spruce needles	-0,18	-0,43	-0,50	-0,36	**0,75**	-0,07	-0,43	0,00	-0,14	0,36	-0,14	0,11	-0,21	0,04	-0,07	0,29	0,25
Birch branches	-0,30	-0,57	**-0,59**	-0,16	0,20	-0,01	-0,44	-0,33	-0,30	0,14	0,51	-0,16	-0,04	-0,06	0,04	0,08	0,51
Birch leaves	-0,33	-0,20	-0,12	-0,08	-0,10	-0,26	-0,19	-0,13	-0,48	**0,62**	-0,05	0,16	0,28	-0,06	0,20	0,04	-0,15
