# Supplementary material for: Bioaccumulation of 137Cs: Vegetation Responses, Soil Interactions and Ecological Implications in the Northern Taiga Ecosystems
Source: Life (Basel). 2025 May 12;15(5):774. doi: 10.3390/life15050774 (PMC12113250; doi:10.3390/life15050774)
Supplement: Supplementary file 1 [file life-15-00774-s001.zip › Supplementary File S5.pdf]

Supplementary File S5.

Comparison of the specific activities of 137Cs in the components of a single plant.

Supplementary S5-1. Comparison using the t-test (for dependent variables).

Index	N	Average	t
Asp of ^{137}Cs in the branches of Scots pine, Bq/kg	12	14,8	**-2,8***
Asp of ^{137}Cs in the needles of Scots pine, Bq/kg		19,9	
Asp of ^{137}Cs in the branches of Norway spruce, Bq/kg	7	11,9	0,1
Asp of ^{137}Cs in the needles of Norway spruce, Bq/kg		11,7	
Asp of ^{137}Cs in the branches of silver birch, Bq/kg	8	7,4	**-4,7**
Asp of ^{137}Cs in the leaves of silver birch, Bq/kg		13,8	
Asp of ^{137}Cs in the branches of northern bilberry, Bq/kg	12	28,5	**-5,8**
Asp of ^{137}Cs in the leaves of northern bilberry, Bq/kg		51,4	
Asp of ^{137}Cs in the branches of bog rosemary, Bq/kg	11	21,8	**-5,2**
Asp of ^{137}Cs in the leaves of bog rosemary, Bq/kg		49,1	

Comparison using the Wilcoxon signed-rank test (for dependent variables)

Index	N	Average	Z
Asp of ^{137}Cs in the branches of European blueberry, Bq/kg	12	53,3	**2,5****
Asp of ^{137}Cs in the leaves of European blueberry, Bq/kg		74,2	

**Critical values of the t-test for n=7, n=8, n=11, and n=12 are |2.4|, |2.3|, |2.2|, and |2.18|, respectively.*

**The value of the Wilcoxon test exceeds the critical value according to Statistica 10.

Supplementary S5-2. Comparison of specific activities of 137Cs between plant components of different species

Comparing Components of Different Plant Species	Average Asp of ^{137}Cs in Component 1, Bq/kg	Average Asp of ^{137}Cs in Component 2, Bq/kg	t
Pine branches — Blueberry branches	14,8	53,3	**-5,6***
Pine branches — Bilberry branches	14,8	28,5	**-2,8**
Pine branches — Bilberry leaves	14,8	51,4	**-7,4**
Pine branches — Pleurozium	14,8	49,4	**-4,7**
Pine branches — Bog rosemary branches	14,8	21,8	**-2,3**
Pine branches — Bog rosemary leaves	14,8	49,1	**-5,1**
Pine branches — Spruce branches	14,8	11,9	1,3
Pine branches — Spruce needles	14,8	11,7	1,4
Pine branches — Birch branches	14,8	7,4	**4,0**
Pine branches — Birch leaves	14,8	14,4	0,2
Pine needles — Blueberry branches	19,9	53,3	**-4,7**
Pine needles — Bilberry branches	19,9	28,5	-1,6
Pine needles — Bilberry leaves	19,9	51,4	**-5,9**
Pine needles — Pleurozium	19,9	49,4	**-3,9**
Pine needles — Bog rosemary branches	19,9	21,8	-0,5
Pine needles — Bog rosemary leaves	19,9	49,1	**-4,2**
Pine needles — Spruce branches	19,9	11,9	**2,3**
Pine needles — Spruce needles	19,9	11,7	**2,4**
Pine needles — Birch branches	19,9	7,4	**4,3**
Pine needles — Birch leaves	19,9	14,4	2,0
Blueberry branches — Bilberry branches	53,3	28,5	**3,1**
Blueberry branches — Bilberry leaves	53,3	51,4	0,2

Comparing Components of Different Plant Species	Average Asp of ^{137}Cs in Component 1, Bq/kg	Average Asp of ^{137}Cs in Component 2, Bq/kg	t
Blueberry branches — Pleurozium	53,3	49,4	0,4
Blueberry branches — Bog rosemary branches	53,3	21,8	**4,2**
Blueberry branches — Bog rosemary leaves	53,3	49,1	0,4
Blueberry branches — Spruce branches	53,3	11,9	**4,6**
Blueerry branches — Spruce needles	53,3	11,7	**4,7**
Blueberry branches — Birch branches	53,3	7,4	**5,9**
Blueberry branches — Birch leaves	53,3	14,4	**5,5**
Bilberry branches — Pleurozium	28,5	49,4	**-2,4**
Bilberry branches — Bog rosemary branches	28,5	21,8	1,2
Bilberry branches — Bog rosemary leaves	28,5	49,1	**-2,6**
Bilberry branches — Spruce branches	28,5	11,9	**2,7**
Bilberry branches — Spruce needles	28,5	11,7	**2,7**
Bilberry branches — Birch branches	28,5	7,4	**3,9**
Bilberry branches — Birch leaves	28,5	14,4	**2,9**
Bilberry leaves — Pleurozium	51,4	49,4	0,2
Bilberry leaves — Bog rosemary branches	51,4	21,8	**5,3**
Bilberry leaves — Bog rosemary leaves	51,4	49,1	0,3
Bilberry leaves — Spruce branches	51,4	11,9	**6,3**
Bilberry leaves — Spruce needles	51,4	11,7	**6,3**
Bilberry leaves — Birch branches	51,4	7,4	**8,0**
Bilberry leaves — Birch leaves	51,4	14,4	**7,4**
Pleurozium — Bog rosemary branches	49,4	21,8	**3,5**
Pleurozium — Bog rosemary leaves	49,4	49,1	0,0
Pleurozium — Spruce branches	49,4	11,9	**3,9**
Pleurozium — Spruce needles	49,4	11,7	**4,0**
Pleurozium — Birch branches	49,4	7,4	**5,1**
Pleurozium — Birch leaves	49,4	14,4	**4,7**
Bog rosemary branches — Spruce branches	21,8	11,9	**2,8**
Bog rosemary branches — Spruce needles	21,8	11,7	**2,9**
Bog rosemary branches — Birch branches	21,8	7,4	**4,7**
Bog rosemary branches — Birch leaves	21,8	14,4	**2,6**
Bog rosemary leaves — Spruce branches	49,1	11,9	**4,3**

Comparing Components of Different Plant Species	Average Asp of ^{137}Cs in Component 1, Bq/kg	Average Asp of ^{137}Cs in Component 2, Bq/kg	t
Bog rosemary leaves — Spruce needles	49,1	11,7	**4,3**
Bog rosemary leaves — Birch branches	49,1	7,4	**5,5**
Bog rosemary leaves — Birch leaves	49,1	14,4	**5,0**
Spruce branches — Birch branches	11,7	7,4	**3,5**
Spruce needles — Birch leaves	11,7	14,4	-1,7
